# OA07.04. Self-care, use of CAM and satisfaction with health care in people with inadequately controlled Type 2 diabetes

**DOI:** 10.1186/1472-6882-12-S1-O28

**Published:** 2012-06-12

**Authors:** R Bradley, K Sherman, S Catz, C Calabrese, E Oberg, D Cherkin

**Affiliations:** 1 Bastyr University, Kenmore, USA; 2 Group Health Research Institute, Seattle, USA; 3 Naturopathic Physicians Research Institute, Portland, USA

## Purpose

To test the hypothesis that people with inadequately controlled Type 2 diabetes (T2D) interested in adjunctive naturopathic care (ANC) may differ in their current self-care behavior and motivation for behavior change compared to those with less interest. We also aimed to measure CAM use and satisfaction with health care.

## Methods

Patients with inadequately controlled T2D from Group Health Cooperative were invited to participate in a telephone survey. The survey queried interest in ANC, current CAM use, and current self-care. Self-care behavior, perceptions about blood sugar, and motivation for behavior change were assessed using the Summary of Diabetes Self-care Activities, Perceptions of Blood Sugar Control and the Readiness Index instruments, respectively. Survey responses were then compared between people who expressed great interest in using ANC services and those who expressed less interest.

## Results

219 of 321 eligible patients (68.5%) completed the survey. Nearly half of the respondents (48%) expressed strong interest in ANC services. Patient demographics, health history, and self-care behaviors did not differ by ANC interest. People interested in ANC were more likely to: have a plan to change self-care (p=0.01), be more determined to succeed in self-care (p=0.007) and have a long-term commitment to change self-care (p=0.02). Use of several CAM therapies was higher in the ANC-interested group, including vitamin and mineral supplements, herbal and nutritional supplements and meditation (p<0.05 for each). Those interested in ANC perceived their current health care as less beneficial for blood sugar control than those less interested (mean response: 5.9 +/- 1.9 vs. 6.6 +/- 1.5, p=0.003).

## Conclusion

People with T2D interested in ANC do not differ in their current self-care, but are more motivated for self-care improvement. Dissatisfaction with current care for T2D may influence their interest in CAM.

